# Tiny but Mighty: A Low Dose of Standardized Dry Grape Extract Potentiates Antioxidant Defenses in Broiler Chickens

**DOI:** 10.3390/antiox15091216

**Published:** 2026-09-21

**Authors:** Paul Engler, Sekhou Cisse, Arkadiusz Matuszewski, Sławomir Jaworski, Agata Lange, Damian Bień, Patrycja Kostusiak, Anna Zalewska, Jakub Urban, Monika Michalczuk, Marta Mendel, Urszula Latek, Joanna Polak, Mohammed El Amine Benarbia

**Affiliations:** 1 Joint Lab ANR FeedInTech (FIT: SONAS/Nor-Feed), 49070 Beaucouze, France; sekhou.cisse@norfeed.net (S.C.); amine.benarbia@norfeed.net (M.E.A.B.); 2 Nor-Feed SAS, 3 rue Amédéo Avogadro, 49070 Beaucouze, France; 3 Department of Animal Environment Biology, Institute of Animal Sciences, Warsaw University of Life Sciences (SGGW), Ciszewskiego 8, 02-786 Warsaw, Poland; arkadiusz_matuszewski@sggw.edu.pl; 4 Department of Nanobiotechnology, Institute of Biology, Warsaw University of Life Sciences (SGGW), Ciszewskiego 8, 02-786 Warsaw, Poland; slawomir_jaworski@sggw.edu.pl (S.J.); agata_lange1@sggw.edu.pl (A.L.); 5 Department of Animal Breeding and Nutrition, Institute of Animal Sciences, Warsaw University of Life Sciences, Ciszewskiego 8, 02-786 Warsaw, Poland; damian_bien1@sggw.edu.pl (D.B.); patrycja_ciborowska@sggw.edu.pl (P.K.); anna_zalewska1@sggw.edu.pl (A.Z.); jakub_urban@sggw.edu.pl (J.U.); 6 Department of Preclinical Sciences, Division of Pharmacology and Toxicology, Institute of Veterinary Medicine, Ciszewskiego 8, 02-786 Warsaw, Poland; marta_mendel@sggw.edu.pl (M.M.); urszula_latek@sggw.edu.pl (U.L.); joanna_polak@sggw.edu.pl (J.P.)

**Keywords:** polyphenols, grape extract, broiler, antioxidant

## Abstract

Oxidative stress can impair poultry health and performance, underscoring the importance of enhancing endogenous antioxidant defenses as a nutritional strategy. Although plant-derived polyphenols have shown antioxidant potential, their efficacy remains inconsistent owing to variability in extract composition and the frequent evaluation of doses exceeding those authorized for commercial use. This study investigated the effects of dietary supplementation with a standardized dry grape extract (SDGE, Nor-Grape^®^, Nor-Feed, France) at an authorized inclusion level (30 mg/kg feed) on growth performance and antioxidant status in broiler chickens under commercial production conditions. A total of 756 one-day-old male Ross 308 broilers were randomly allocated to a control diet (CTL, n = 392) or the same diet supplemented with grape extract (NG, n = 364) for 35 days. Growth performance was recorded throughout this study, and blood samples collected on days 11 and 35 were analyzed for total antioxidant capacity (TEAC), lipid peroxidation (TBARS), and the activities of the endogenous antioxidant enzymes catalase (CAT), superoxide dismutase (SOD), and glutathione peroxidase (GSH-Px). Compared with controls, NG birds exhibited greater final body weight (+71.9 g; *p* < 0.001) and improved FCR during the finisher phase (*p* < 0.05). Supplementation significantly enhanced systemic antioxidant capacity and endogenous antioxidant enzyme activities while reducing lipid peroxidation, indicating improved oxidative status. These findings demonstrate that SDGE supplementation at commercially authorized inclusion levels effectively enhances antioxidant defenses and improves production performance in broiler chickens.

## 1. Introduction

With an estimated total production close to 140 million tons in 2025, broiler chickens are one of the most produced animal species on the planet [1]. This demand, accompanied by increased genetic selection to create fast-growing birds, ready to butcher within 35 days, has also progressively changed their metabolism towards an increased metabolic activity [2]. These increased metabolic rates have led to increased levels of Reactive Oxygen Species (ROS, more commonly known as free radicals) by the mitochondria, triggering increased metabolic issues related to oxidative stress mismanagement [3]. Indeed, excessive ROS production can lead to overwhelming antioxidant defenses, resulting in the reaction of these harmful species with cellular components and the generation of oxidative damage. This phenomenon is called oxidative stress [4].

While the cost of oxidative stress mismanagement for the broiler industry has not yet been estimated precisely, it is known to have negative consequences on several key economic and zootechnical indicators of poultry production, such as feed conversion rate (FCR) and growth performance [3,5], mortality [6], meat quality [7] and sensitivity to diseases through immunosuppression [8].

To counteract this phenomenon, dietary nutrients playing a role in the antioxidant system have been used for decades. The main ones are synthetic vitamin E [9] and selenium [10]. Recent studies have, however, shown that synthetic vitamin E (alpha-tocopherol acetate) might have limitations when used in young chicks [5], and only a fraction of the stereoisomers are actually bioavailable due to the incompatibility of the SS-forms with the active transporters responsible for their uptake [11]. Selenium, on the other hand, faces limitations in terms of regulatory maximum inclusion levels. While the use of organic selenium, with higher bioavailability, allows for better uptake, it still faces regulatory limitations and thus, offers reduced flexibility [10]. To overcome these challenges, the use of a new class of antioxidant compounds has been increasingly studied over the past decades in animal nutrition, phenolic compounds, also known as polyphenols [12,13].

These compounds constitute a very large family, estimated at around 8000 known compounds to date [12]. This generic term thus hides a very wide diversity in terms of structure and thus bioavailability [14]. To address these challenges, the poultry industry consequently needs existing solutions that are readily available (from both a regulatory and a commercial point of view) to be able to implement them rapidly. Unfortunately, published studies too often use very high doses of products that are either not allowed by regulations, are detrimental to performance, or are just economically not relevant. The lack of qualification in the composition of the solution tested also represents a bias in the generalization of the results [15]. Ringseis et al. [15], for instance, recently published a review indicating potential ranges of use of polyphenol-containing grape by-products, indicating that grape extracts’ range for performance enhancement would be around 10 to 500 mg/kg diet [16], which exceeds, for most of this range, authorized levels in feedstuffs.

The aim of the present study was to evaluate the effect of a dietary supplementation of broilers with a low dose of commercial standardized dry grape extract (SDGE, readily usable under the EU regulation [17]) on their growth performances and antioxidant parameters.

## 2. Materials and Methods

### 2.1. Ethical Statement

Ethical review and approval were waived by the II Local Ethics Committee for Animal Experiments at Warsaw University of Life Sciences (WULS, Warsaw, Poland). Following review of the study protocol, the Committee concluded that the activities performed in this study did not constitute an experimental procedure within the meaning of the Polish Act of 15 January 2015 on the Protection of Animals Used for Scientific or Educational Purposes. The broiler chickens were maintained under standard husbandry conditions, and biological samples were collected post mortem. No procedures involving pain, suffering, distress, or lasting harm were performed on the broiler chickens. Therefore, ethical approval was not required under the above-mentioned Act (official letter dated 26 April 2023).

### 2.2. Experimental Design

The trial was carried out at the WULS Agricultural Experimental Station. In total, 756 healthy Ross 308-day-old male chicks were randomly allocated to dietary treatments in a windowless farm in 27 pens (1.45 × 1.45 m, 28 birds per pen, maximal stocking density 33 kg/m^2^) within thermostatically controlled conditions. The pens included in the present study were then assigned to the CTL or NG treatment according to a predefined spatial arrangement, with 14 pens assigned to CTL and 13 pens to NG. Pens from both treatments were distributed throughout the experimental facility to avoid clustering of either treatment in a particular area of the building. They were then anonymized so that operators would not know the corresponding groups. The average initial body weight of day-old chicks was 45.4 g ± 2.7 g. The lighting program was as recommended by Ross: 23 h of daylight from D0 to D7, then 19 h of daylight until the end of the trial period. Each pen was equipped with a tube feeder and 2 bell drinkers during the first week of life. After that, bell drinkers were replaced by automatic nipple drinkers. Straw pellets were used as litter, and birds had ad libitum access to feed and water throughout the trial. Chickens were reared to 35 days of age starting at 34° at chick placement and gradually reduced to 25.5 °C by 25 days of age, where this temperature was maintained thereafter. Chickens were fed with a three-phase system: starter (1–10 day), grower (11–27 day), and finisher (28–35 day) (Table 1).

The birds were divided into 2 homogeneous groups depending on whether or not they received a commercial standardized dry grape extract (SDGE, Nor-Grape^®^ 80, Nor-Feed, France). The SDGE is a mixture of grape seed extracts and grape skin extracts obtained by water extraction and spray-drying. It is rich in polyphenols (80% total polyphenols, 60% procyanidins, and 0.75% anthocyanins). Its main phenolic compounds identified were previously reported [18]: catechin (0.84 g per 100 g), epicatechin (0.77 g per 100 g), procyanidin dimer B1 (0.68 g per 100 g), procyanidin dimer B2 (0.49 g per 100 g), epicatechin-O-gallate (0.097 g per 100 g), and gallic acid (0.36 g per 100 g). The control group (CTL, 14 pens with 392 birds) was fed the standard diet, and the supplemented group (NG, 13 pens with 364 birds) was fed the control diet with 30 mg of SDGE per kilogram of feed. The application of the product was in the starter, grower, and finisher diets, that is, throughout the entire period of chicken rearing.

The inclusion rate of 30 mg/kg feed was the targeted inclusion rate. However, like most natural extracts, due to the large diversity of phenolic compounds in the additive and the presence of native phenolic compounds in the raw materials of the diet, the development of a specific quantification of the additive in the diet is necessary for such a small inclusion rate. To address this problem, a method based on a specific sample preparation and UHPLC-MS/MS has been developed and validated by Nor-Feed for this specific grape extract and has previously been reported [19]. A 500 g sample of each diet was taken at the experimental farm on the first day of each period and sent for analysis to Nor-Feed to quantify the amount of SDGE in the trial diets. The feed samples from this experiment reported the absence of SDGE in all CTL diets and 26.9 mg/kg starter feed, 24.3 mg/kg grower feed, and 18.1 mg/kg finisher feed, expressed in Nor-Grape^®^ 80 equivalent.

Feed labeling was anonymized so experimental farm workers were not aware of the group allocated to each pen. Animal health conditions were monitored on a daily basis to detect any potential abnormality in the general health status of the birds and the need for veterinary intervention. Mortality was also routinely monitored for each pen. Feed samples of each production period and group were retained at production and blindly analyzed by an external laboratory for proximate composition analysis (dry matter, crude protein, ether extract, crude fiber, ash). The diet composition for the different stages and groups is presented in Table 2.

### 2.3. Zootechnical Parameters

Body weight per bird and feed intake (FI) were measured in each dietary transition (d 10, d 27, and d 35). Feed conversion ratio (FCR) was also calculated at d 10, d 27, and d 35, and mortality was recorded daily. Based on the parameters monitored, the European Efficiency Index (EEI) was calculated using the following formula:

EEI = (average body weight (kg) × survival rate (%))/(rearing days × feed consumption per kg body weight)

### 2.4. Blood Antioxidant Parameters

On D11 and D35, thirty chickens from each group with body weights similar to the group average were randomly selected (two to three per pen) for blood collection. Blood samples were collected post mortem after decapitation of the selected birds in heparin tubes and immediately placed in an insulated polystyrene container with ice for transport to the laboratory. Plasma was separated from whole blood by centrifugation at 3000× *g* for 10 min at 4 °C. Following centrifugation, plasma samples were visually inspected for hemolysis using a hemolysis reference color palette [20]. Following separation and assessment, plasma was aliquoted and stored at −20 °C until analysis. All biochemical analyses were performed within 7 days of blood collection. Individual aliquots were thawed only once, immediately prior to analysis, thereby avoiding repeated freeze–thaw cycles. Each biological sample was analyzed in technical duplicate. Selected redox state markers were determined in plasma using commercially available kits (adapted for plasma samples and activity- or reaction-based reliance on chemical or enzymatic reactions) and following the manufacturers’ procedures: glutathione peroxidase (GSH-Px, ab102530, Abcam, Cambridge, UK); superoxide dismutase (SOD, MAK379, Sigma Aldrich, Darmstadt, Germany); catalase (CAT, 219265, Merck, Darmstadt, Germany); Trolox Equivalent Antioxidant Capacity (TEAC, MBS169313, MybioSource, Vancouver, Canada); and Thiobarbituric acid reactive substances TBARS via the measure of malondialdehyde (MDA, MAK085, Sigma Aldrich, Darmstadt, Germany).

### 2.5. Statistical Analysis

The statistical unit for the zootechnical data was the pen. Statistical analyses were performed using Student’s test (*t*-test) after verification of the normality of the data using the Shapiro–Wilk normality test. Non-normal data were analyzed using the Mann–Whitney U test. The statistical threshold for significance was set at *p* < 0.05. When a stronger significance level was observed, such observations were detailed (e.g., *p* < 0.001).

The statistical unit for the blood analyses was the individual chicken. Blood data were analyzed using the following linear mixed-effects model, including the fixed effects of treatment, sampling day, and their interaction, with the pen included as a random effect:

$$Y_{ijkl}=\mu +{Group}_{i}+Day_{j}+{\left(Group\times Day\right)}_{ij}+Pen_{k}+\varepsilon_{ijkl}$$

Estimated marginal means were used for post hoc comparisons when significant main effects or interactions were detected. The collected data were processed using R (version 4.6.1).

## 3. Results

### 3.1. Zootechnical Performances

In general, animal health conditions were good throughout the trial. No significant difference was observed between dietary treatments in terms of mortality. Growth performance of both groups was relatively similar throughout most of the growing period (Table 3). At the end of the production phase, however, on D35, broiler chickens supplemented with Nor-Grape were significantly bigger on average than chickens from the CTL group (+71.9 g, *p* < 0.001). Furthermore, no differences were observed in mortality between groups, and FCR did not differ significantly between treatments during the starter and grower phases, whereas the finisher FCR was significantly improved in the supplemented group.

### 3.2. Blood Antioxidant Parameters

Analyses to determine plasma antioxidant potential (Table 4) were conducted on D11 and D35. Overall, the day of sampling had a significant effect on all parameters studied (*p* < 0.05) except MDA. The supplementation significantly influenced TEAC, CAT and SOD and tended to impact MDA too. The interaction between both day and group also significantly influenced GSH-PX.

The overall antioxidant capacity (TEAC) of NG chickens supplemented was significantly increased as soon as D11 (+28%, *p* < 0.001). While it increased significantly in both groups over time (*p* < 0.001), it remained significantly higher on D35 in the NG group (+4.3%, *p* < 0.05).

Endogenous antioxidant enzymes were also positively modulated by the supplementation. On D11, CAT and SOD were significantly increased in the NG group (+27.8%, *p* < 0.01; +8.3%, *p* < 0.01, respectively). Whilst all endogenous antioxidant defenses increased over time in both groups (*p* < 0.05), on D35, NG broilers showed increased levels of CAT, SOD and GSH-Px compared to CTL ones (+15.7%, *p* < 0.05; +9.0%, *p* < 0.001; and +16.7%, *p* < 0.001, respectively).

On D11, NG chickens tended to have lower oxidative damages than CTL ones (−7.7%, *p* < 0.10). Interestingly, while no significant difference was observed between D11 and D35 for the CTL birds (*p* > 0.05), it was significantly reduced in NG birds on D35 (−14.9%, *p* < 0.01).

## 4. Discussion

### 4.1. Zootechnical Performances

Oxidative stress impact on livestock, and more specifically on broiler chickens, has long been studied and has been documented to negatively impact performance such as growth rate, FCR, or body weight [3,4]. Recent research, for instance, showed that increasing oxidative pressure impairs broilers’ FCR, with a growing effect as the birds age [21]. In the current study, NG chickens evidenced a significantly higher final body weight and a lower FCR during the finisher phase. This, combined with the significantly increased antioxidant defenses and significantly reduced oxidative damages, could be explained by a reduction in the oxidative pressure on the NG chickens, thus alleviating the harmful effects of oxidative stress on the broilers’ growth performances. These findings concur with previous studies, where grape by-products, rich in polyphenols, were used in fast-growing broiler chickens [16]. The authors of this review indeed reported that the use of grape pomace, grape seed or grape seed extract, in most cases, increased the body weight of chickens and/or reduced FCR. It is, however, important to underline that these reported studies were carried out with a wide range of inclusion rates and varying compositions, making it difficult to reach a simple conclusion. For instance, a study evaluated the effect of a dietary supplementation of grape seed extract containing 40% total polyphenols with doses ranging from 125 mg/kg to 2000 mg/kg, equivalent to 50 mg to 800 mg of total grape polyphenols per kg of complete feed, but did not report any effect on growth performances at the end of the study [22]. Comparatively, in the present trial, using 30 mg/kg of an 80% total polyphenol SDGE meant that the NG group supplementation was at 24 mg of total grape polyphenols per kilogram of complete feed. Despite being more than half the level of the previously discussed study, this lower dose did result in an improvement in the broilers’ growth performances. Similarly, in another study where chickens were supplemented with 1000 mg/kg of a grape seed extract containing 37.3% total condensed tannins, no effect on performances was reported either, despite it providing 373 mg/kg of grape polyphenols [23]. Condensed tannins are a subclass of phenolic compounds that can vary greatly in terms of size, impacting their bioavailability [24,25]. Thus, the lack of effect on growth performance in the discussed study, despite providing 20.7 times more procyanidins than the present trial, might be linked to the profile of these compounds, underlining the fact that all grape extracts might not be equivalent in terms of activity. Furthermore, the use of a grape seed extract containing 80.5% total polyphenols (similar to the SDGE used in this study) at an inclusion rate of 200 and 400 mg/kg did not show any effect on performance at 400 mg/kg but did improve average daily gain and FCR at 200 mg/kg [26]. Finally, a previous study on the SDGE used in this experiment was also carried out on broiler chickens at inclusion rates ranging from 25 to 5000 mg/kg but did not show any improvement in broilers’ performances at 21 days of age [27]. They, however, reported a significant improvement in the crude protein’s apparent ileal digestibility (AID) in the 25 mg/kg group (+2%) as well as that of the glycine. In the present study, 30 mg/kg of this SDGE resulted in improved final body weight and of the finisher period FCR. The improvements in the crude protein AID at 25 mg/kg may well have been at play at 30 mg/kg and could potentially explain the better performance observed here. Interestingly, Chamorro et al. [27] reported that this effect then disappeared when inclusion rates increased, pointing out the fact that there may well be a “sweet spot” of the level of inclusion to get the maximum effect of this supplementation and that very high levels would be too much for it. This could also explain why studies with much higher inclusion rates, discussed previously, did not produce the same positive effects on broilers’ performance.

### 4.2. Antioxidant Defenses

Polyphenols have long been studied since their discovery under the name “vitamin P” in the 1930s [28]. A more important focus has been placed on the study of their physiological antioxidant properties in many species since the end of the last century [14]. Their application in livestock and more particularly in poultry has been widely studied over the last two decades, showing their beneficial role in the antioxidant defense system [29,30,31]. More particularly, due to the increased focus on circularity and the large amount of available raw material worldwide, grape polyphenols originating from the wine industry by-products have been the focus of some very advanced research to evaluate their benefits in poultry, with findings evidencing the physiological antioxidant function of grape polyphenols in these species [16,24,31].

Whilst a previous study on the current grape extract, carried out at 25 mg/kg inclusion rate, did not provide information on the antioxidant parameters [27], the present results showed a rapid response of the NG birds’ antioxidant system to the 30 mg/kg supplementation at D11 for the total antioxidant defenses (TEAC), as well as for some endogenous antioxidant enzymes (SOD and CAT) and a tendency to reduce the oxidative damages (MDA). Interestingly, long-term supplementation resulted in significant improvements in all antioxidant parameters: increased total defenses, higher specific endogenous defenses, and reduced lipid oxidation. These findings are consistent with the vast majority of studies on the use of grape polyphenols in livestock and, more specifically, in poultry, showing the interest of these natural compounds in stimulating the antioxidant defense system. Remarkably, this grape extract in its rumen-protected form was also recently shown to modulate gene expression in the leucocytes of heat-stressed dairy cattle, leading to the induction of endogenous antioxidant defenses as observed here [32], therefore suggesting that the increase observed in the present experiment may be ubiquitous and linked to genomic modulation to increase the quantity of these enzymatic defenses. Furthermore, the current supplementation (30 mg/kg of Nor-Grape^®^ grape extract) was also recently reported to beneficially influence the mitochondrial function in pullets [33] and trigger several metabolic pathways, suggesting that these grape polyphenols might not just be simple antioxidants but actually act as metabolic modulators. The Nrf2/Keap1/ARE pathway plays a crucial role in the management of the antioxidant response of poultry [4] and leads to the increased production of endogenous antioxidant defenses such as SOD, CAT, and GSH-Px. This pathway has been shown to be modulated by some specific phenolic compounds [34], in particular, grape polyphenols [31]. In the present study, despite its low inclusion rate, the NG supplementation led to a significant modulation of all three endogenous antioxidant enzymes, resulting in an increased level of these defenses compared to the CTL group. This therefore underlines that the polyphenols at play may have triggered these signalization pathways, even at such low inclusion rates, as previously reported in ruminants [32], thus concurring with the existing evidence of such a mode of action for specific grape polyphenols.

Interestingly, in their recent review on the use of grape by-products, Ringseis et al. [15] noted that the vast majority of studies showed a beneficial effect on the antioxidant system but reported that only two studies reported no effect or inconsistent effects on oxidative stress and antioxidant status. No effect of dietary supplementation with 100 mg/kg or 200 mg/kg grape seed extract for 6 weeks was reported in supplemented broilers, with the authors of the review hypothesizing that one explanation for the lack of effect might be the relatively low dose used compared to the majority of studies with positive outcomes, where higher inclusion rates were used. However, the present study challenges this idea, showing that a 30 mg/kg inclusion rate can elicit a positive response at the antioxidant defense level. This underlines that a qualitative approach might be at play, with some specific polyphenols being more active than others in activating pathways that are behind the observed effects, thus stimulating the antioxidant system.

Comparatively, another study with a less concentrated grape extract (125 mg/kg of grape extract containing 40% total polyphenols, equivalent to 50 mg/kg supplemented polyphenols) showed enhanced reduced GSH content and lower TBARS values in broilers [22], concurring with the present study but corresponding to double the quantity of total polyphenols compared to the current work (30 mg/kg of a grape extract containing 80% total polyphenols, equivalent to 24 mg/kg supplemented polyphenols). Conversely, another supplementation study with a grape extract containing a similar amount of total polyphenols (80.5%) showed comparable results on total antioxidant capacity and CAT increase and oxidized lipid reduction at both 200 mg/kg and 400 mg/kg. However, an increase in SOD was only noted at 200 mg/kg, not 400 mg/kg, and GSH-Px increased only at 400 mg/kg, not 200 mg/kg. Comparatively, the present supplementation providing a similar phenolic content in the additive was able to achieve a global stimulation of the antioxidant system at a dose virtually 10 times smaller.

The inclusion rate might, nevertheless, not be the only aspect to take into consideration, as underlined in Andrés’ recent work [25], a strong discrepancy between polyphenol bioavailability affects the generalization of results. As underlined in another recent study [15], this key aspect limits the comparability of the results across studies, especially when they only rely on the plant species rather than the composition of the solution in terms of nature and quantity of phenolic compounds. This might actually be the prime reason for the divergence in responses between high inclusion rate and no response vs. low inclusion rate and high response. For instance, the apparent digestibility of the phenolic compounds present in the studied grape extract was shown to be superior to 80% [35]. Thus, more than the quantitative approach, for better stimulation of the antioxidant defenses with grape polyphenols, a qualitative approach with bioavailability in mind might be the key to explain why such a much lower dose could provide similar to higher results than classically higher inclusion rates. This could be a source of explanation regarding the visible response as early as at D11 too.

Finally, NG supplementation of broiler chickens at a low theoretical dose of 30 mg/kg led to an increase in the global antioxidant defense system as well as the stimulation of the production of endogenous antioxidant defenses. This better protection translates into lower oxidative damages at the cellular level. Excessive oxidative pressure has been linked to decreased growth performances [3,5]. In the present work, reduction in oxidative damage was concomitant with better growth at the zootechnical level, with heavier final weight at D35. This suggests that the better antioxidant protection provided with the NG supplementation may lead to better growth performance due to a lower impact of the oxidative pressure on the bird. Interestingly, the quantification of the additive in the trial feeds revealed an actual inclusion rate inferior to the targeted 30 mg/kg, suggesting that the effects observed could consequently be attributed to an even lower dose.

The present study, however, only studied a single low dose in all-male broilers, and further research on dose–response around this range of inclusion might be necessary to assess the optimal level of SDGE that would represent the best compromise between efficacy and regulatory possibilities in both normal conditions (as evaluated here) and in challenging conditions.

## 5. Conclusions

Grape polyphenols have demonstrated their undeniable positive effects in providing better antioxidant protection to livestock, but discrepancies remain regarding the understanding of the inclusion level to be used, both in accordance with scientific evidence and regulatory adequacy. In the present study, we demonstrated that a specific standardized dry grape extract, even at a low inclusion rate of 30 mg/kg, can still provide marked physiological antioxidant protection, both on global antioxidant defenses and on the stimulation of endogenous antioxidant defense production. This resulted in lower oxidative damage and higher final live weight. These findings underline that more than a quantitative approach, future research should focus on a qualitative approach to better understand which levels could be used to optimize the formulation of livestock diets.

## Figures and Tables

**Table 1:** Feed composition.

Components	Starter (1–10 d) (kg)	Grower (11–27 d) (kg)	Finisher (28–35 d) (kg)
Corn 8.2%	20	20	20
Wheat 11.5%	41.741	42.513	35.751
Triticale 10.5%	-	-	10
Soybean meal 46%	28.159	25.369	21.554
Rapeseed meal 35%	4	5	5
Soybean oil	2.315	4.013	4.773
L-lysine 78%	0.419	0.36	0.347
DL-methionine 98%	0.311	0.196	0.218
L-threonine 98%	0.141	0.091	0.108
Limestone	1.47	1.133	1.121
Monocalcium phosphate	0.843	0.723	0.522
Salt	0.324	0.326	0.329
Vitamin–mineral PX ^1^	0.25	0.25	0.25
Ronozyme WX	0.012	0.012	0.012
Phytase	0.015	0.015	0.015
Total (kg)	100	100	100
ME (kcal/kg)	2950	3100	3150
Crude fiber (%)	3.43	3.43	3.31
Crude protein (%)	21.5	20.5	19
Lysine (g/kg)	13.5	12.5	11.5
Methionine (g/kg)	6.21	5	5.02
Met. + Cys. (g/kg)	10	8.7	8.5
Threonine (g/kg)	9	8.2	7.8
Tryptophan (g/kg)	2.55	2.43	2.23
Arginine (g/kg)	13.35	12.68	11.58
Calcium (g/kg)	10	8.5	8
Phosphorus (g/kg)	6.87	6.55	5.89
Avail. Phosphorus (g/kg)	4.8	4.5	4
Sodium (g/kg)	1.5	1.5	1.5
Chloride (g/kg)	3.3	3.18	3.11
	^1^ Provided per kilogram of diet: vitamin A (E 672): 10.000 IU; vitamin D3 (E 671): 4.000 IU; vitamin E (a-tocopherol): 15.0 mg; vitamin K3: 3.0 mg; vitamin B1: 2.0 mg; vitamin B2: 5.0 mg; vitamin B6: 4.0 mg; vitamin B12: 11.0 µg; nicotinic acid: 40.0 mg; calcium pantothenate: 12.0 mg; folic acid: 2.0 mg; biotin: 0.18 mg; Cu: 8.0 mg; Fe: 50.0 mg; I: 2 mg; Mn: 70.0 mg; Se: 0.15 mg; Zn: 80.0 mg.	^1^ Provided per kilogram of diet: vitamin A (E 672): 10.000 IU; vitamin D3 (E 671): 4.000 IU; vitamin E (a-tocopherol): 15.0 mg; vitamin K3: 3.0 mg; vitamin B1: 2.0 mg; vitamin B2: 5.0 mg; vitamin B6: 4.0 mg; vitamin B12: 11.0 µg; nicotinic acid: 40.0 mg; calcium pantothenate: 12.0 mg; folic acid: 2.0 mg; biotin: 0.18 mg; Cu: 8.0 mg; Fe: 50.0 mg; I: 2 mg; Mn: 70.0 mg; Se: 0.15 mg; Zn: 80.0 mg.	^1^ Provided per kilogram of diet: vitamin A (E 672): 10.000 IU; vitamin D3 (E 671): 4.000 IU; vitamin E (a-tocopherol): 10.0 mg; vitamin K3: 3.0 mg; vitamin B1: 2.0 mg; vitamin B2: 5.0 mg; vitamin B6: 4.0 mg; vitamin B12: 11.0 µg; nicotinic acid: 40.0 mg; calcium pantothenate: 12.0 mg; folic acid: 2.0 mg; biotin: 0.18 mg; Cu: 8.0 mg; Fe: 50.0 mg; I: 2 mg; Mn: 70.0 mg; Se: 0.15 mg; Zn: 80.0 mg.

**Table 2:** Proximal analysis data from the different feeds used in the trial.

	Control			Nor-Grape		
Ingredients (g/100 g)	Starter	Grower	Finisher	Starter	Grower	Finisher
**Dry matter**	93.2	93.2	92.9	93.4	93.8	93.0
**Crude protein**	20.1	19.3	18.7	20.2	19.1	18.2
**Ether extract**	3.60	5.75	5.70	3.72	5.42	5.82
**Crude fibre**	3.11	2.51	2.75	3.00	2.35	2.62
**Ash**	4.84	4.78	4.48	4.84	4.70	4.66

**Table 3:** Chicken growth results in the control (CTL) and supplemented (NG) groups.

Parameters	CTL	NG	*p*-Value
**Body weight [g]:**			
**D1**	45.6 ± 0.4	45.4 ± 0.5	ns
**D10**	186.1 ± 3.1	187.2 ± 4.2	ns
**D27**	1620.8 ± 29.9	1628.5 ± 18.2	ns
**D35**	2304.5 ± 34.8 ^a^	2376.4 ± 35.1 ^b^	*p* < 0.001
**FI starter [g/bird]**	170.8 ± 11.1	175.4 ± 8.1	ns
**FI grower [kg/bird]**	1.96 ± 0.2	1.95 ± 0.1	ns
**FI finisher [kg/bird]**	1.04 ± 0.1	1.08 ± 0.1	ns
**FCR starter [kg kg^−1^]**	1.24 ± 0.09	1.28 ± 0.09	ns
**FCR grower [kg kg^−1^]**	1.38 ± 0.08	1.38 ± 0.10	ns
**FCR finisher [kg kg^−1^]**	1.51 ± 0.05 ^a^	1.45 ± 0.07 ^b^	*p* < 0.05
**Overall feed intake [kg/bird]**	3.17 ± 0.21	3.21 ± 0.19	ns
**Overall FCR**	1.41 ± 0.06	1.39 ± 0.08	ns
**Mortality [%]**	3.1 ± 3.7	2.7 ± 3.0	ns
**EEI [points]**	455.5 ± 33.9	476.2 ± 35.6	ns

^a,b^: Different superscripts within the same row indicate a significant difference between groups. The statistical significance is indicated in the furthest column on the right-hand side of the table.

**Table 4:** Chickens’ antioxidant parameters in the control (CTL) and supplemented (NG) groups at D11 and D35 of the trial. Values are presented as mean ± standard deviation.

Day	D11		D35		*p*-Value		
Parameters	CTL	NG	CTL	NG	Group	Day	Group × Day
**TEAC** **[μM]**	66.6 ^ax^ ± 13.1	85.5 ^bx^ ± 18.5	272.6 ^ay^ ± 15.7	281.7 ^by^ ± 8.8	*p* < 0.001	*p* < 0.001	*p* < 0.10
**CAT [nmol/min/mL]**	1.83 ^ax^ ± 0.65	2.34 ^bx^ ± 0.88	2.39 ^ay^ ± 0.64	2.77 ^by^ ± 0.61	*p* < 0.01	*p* < 0.01	0.608
**SOD** **[%, inhibition rate]**	70.3 ^ax^ ± 9.2	76.1 ^bx^ ± 8.8	83.1 ^ay^ ± 6.4	90.6 ^by^ ± 3.7	*p* < 0.01	*p* < 0.001	0.542
**GSH-Px** **[U]**	1736 ^x^ ± 315	1839 ^x^ ± 259	1930 ^ay^ ± 352	2253 ^by^ ± 263	0.199	*p* < 0.05	*p* < 0.05
**MDA** **[nmol/mL]**	878 ^Ax^ ± 164	810 ^Bx^ ± 152	829 ^ax^ ± 191	706 ^by^ ± 87	*p* < 0.10	0.219	0.325

^A,B^: Different superscripts within the same date indicate a tendency to differ between groups (*p* < 0.10) for the parameter observed. ^a,b^: Different superscripts within the same date indicate a significant difference between groups (*p* < 0.05) for the parameter observed. ^x,y^: Different superscripts within the same group indicate a significant difference between dates (*p* < 0.05) for the parameter observed. The values indicated in the columns on the right-hand side of the table correspond to the statistical significances of the linear mixed model for the effect of the group, of the day and of their interaction on the different parameters evaluated.

## Data Availability

The data that support the findings of this study are available on request from the corresponding author (P.E.).
